# Implementing Evidence‐Based Somatosensory Rehabilitation After Stroke: Reported Practice, Documented Practice, and Barriers

**DOI:** 10.1155/oti/6622035

**Published:** 2026-09-15

**Authors:** Anna Joy, Katherine E. Harding, Leeanne M. Carey

**Affiliations:** ^1^ Occupational Therapy, School of Allied Health, Human Services and Sport, La Trobe University, Bundoora, Victoria, Australia, latrobe.edu.au; ^2^ Occupational Therapy Department, Monash University Peninsula Campus, Frankston, Victoria, Australia; ^3^ Eastern Health, Melbourne, Australia, easternhealth.org.au; ^4^ Neurorehabilitation and Recovery, The Florey, Melbourne Brain Centre, Austin Campus, Heidelberg, Victoria, Australia

## Abstract

**Introduction:**

Occupational therapists play a key role in assessing and treating somatosensory impairments post stroke including proprioception (limb position sense), touch, and temperature discrimination. Although research evidence and clinical guidelines offer strategies for assessing and treating this condition, there is limited information on how well these strategies are being implemented in routine practice. We aimed to describe the current practice of occupational therapists in somatosensory assessment and treatment of the upper limb of stroke survivors and to understand barriers and enablers to evidence‐based care.

**Methods:**

This observational study encompassed (1) a cross‐sectional survey measuring self‐reported use of somatosensory assessment and treatment strategies and perceived barriers to implementation and (2) a medical file audit to report on the use of somatosensory assessment and treatment with stroke patients. Data were analyzed descriptively, with enablers and barriers to evidence use classified using the three domains of the Capability, Opportunity, Motivation–Behavior (COM‐B) change model.

**Results:**

Of 50 files audited, 26 patients had documented upper limb impairment. Of these, 54% had sensation assessed. Treatment strategies were documented for 7 out of 12 patients with identified impairment. These findings aligned with therapist self‐report, indicating that most clinicians used sensory assessment and treatment strategies less than once per month. Therapists reported capability (lack of skills and skill confidence) and opportunity (lack of time and opportunity to practice skills) as barriers to evidence‐based practice. Occupational therapists expressed motivation to change and improve their skills in evidence‐based sensory assessment and treatment.

**Conclusion:**

Our findings highlight ongoing gaps between evidence and practice. Therapists report being motivated to provide evidence‐based care but require support to overcome barriers, such as training and access to resources. Medical record audits aligned with survey findings; however, there were differences between reported and documented use and scope of sensory assessment and treatment in practice.

## 1. Introduction

Stroke impacts one in four people during their lifetime [1] with over 445,000 Australians living with ongoing impairment because of stroke [1, 2]. Damage to different parts of the brain can lead to functional impairments for the stroke survivor, including impaired body sensation (somatosensation). Somatosensory function describes a range of body sensory abilities including proprioception, touch, pain, and temperature detection and discrimination [3]. Impairment of these functions is common after stroke, occurring in approximately one in two survivors of stroke, and commonly involves loss of discriminative functions such as touch and proprioception [4, 5]. Impaired somatosensory functions are correlated with lower levels of participation in activities of daily living and poorer outcomes following rehabilitation [6–8].

Effective management of somatosensory impairment after stroke involves the two distinct elements of assessment and treatment. A range of assessment tools exist to assess somatosensory function after stroke [9, 10], including clinical screening tools and quantitative evidence‐based tools such as the Wrist Position Sense Test and the Tactile Discrimination Test [11, 12]. These quantitative measures have been shown to be valid, reliable and have normative standards [11–14]. Effective use of evidence‐based tests requires therapists to be aware of the domain of sensation that they are attempting to assess, know the appropriate assessment to select, have the skills to administer the test, and have the knowledge to interpret the findings [15]. Once sensory loss has been identified through assessment, effective treatment techniques, such as sensory discrimination training, proprioceptive training, and mirror therapy, are indicated [16–20]. Effective, evidence‐based identification and management of somatosensory deficits after stroke is critical, given the association between somatosensation and participation in daily activities [6, 7].

Research evidence and current clinical stroke guidelines in Australia are only valuable if they are being used by therapists [18]. Implementation of evidence‐based practice across the breadth of stroke rehabilitation has been found to be variable [21–23]. In somatosensory rehabilitation, Pumpa and colleagues [24] identified evidence‐practice gaps in both assessment and treatment in Australian rehabilitation settings, with barriers including a lack of knowledge of research findings and treatments, a lack of time and a lack of resources. After 8 years, a study of 85 Australian occupational therapists working in acute care found that nonstandardized assessments were being used by most therapists with a lack of time cited as a barrier to evidence‐based practice [25], suggesting the persistence of evidence practice gaps in this field. Although these studies draw attention to an important issue, they rely only on subjective reporting of therapists, which can be subject to recall or social desirability biases. They do not report on documented measures of the assessments and interventions actually received by patients.

To address evidence‐to‐practice gaps in occupational therapy, it is important to understand the reasons that they persist and design effective implementation strategies. Theoretical frameworks can help to identify facilitators and barriers to the implementation of evidence‐based practice in clinical settings. For example, the Capability, Opportunity, Motivation–Behavior (COM‐B) framework is based on a theory of behavior change and provides an opportunity to consider barriers to the implementation of evidence‐based practice across three domains: Capability refers to both psychological capability (knowledge, understanding, and cognitive skills) and physical capabilities (such as stamina or motor skills); Opportunity includes the environmental context and resources and social influences; and Motivation includes factors such as intentions, plans, and habits [26, 27]. Viewing potential disparities between evidence and practice through the lens of a behavior change framework has been previously shown to highlight direction for future interventions to address these gaps to enhance patient care [28].

This paper extends on previous research by bringing together clinician perceptions of their somatosensory assessment and treatment practices with documentary data from medical files. Specifically, the aims of this study were (1) to describe the current practice of occupational therapists in somatosensory assessment and treatment of the upper limb at a large metropolitan health service and (2) to identify barriers and enablers to implementation of evidence‐based somatosensory assessment and treatment for occupational therapists within the COM‐B domains of capability, opportunity, and motivation [27].

## 2. Method

### 2.1. Design

An observational study was conducted at a large health network, with data collected from two sources. First, a cross‐sectional survey of occupational therapists employed at the health network and working in stroke care was conducted. The survey included self‐reported use of somatosensory measures and treatment strategies for patients after stroke. The survey also aimed to explore factors that may represent enablers or barriers to use of evidence within the COM‐B domains of capability, opportunity, and motivation [27]. Second, a medical file audit was used to triangulate the self‐report data from occupational therapists with evidence of the application of somatosensory assessment and treatment strategies with patients treated at the health service. Data from the survey and file audit were designed to be complementary to permit comparison of findings using self‐report (survey) and written documentation in patient files. Our design was site‐specific, as the intent was to obtain data on self‐reported practice and documented file audit as a baseline for a site‐specific implementation study on evidence‐based sensory rehabilitation. Ethical approval for this study was granted by Eastern Health Human Research Ethics Committee and the La Trobe University Human Research Ethics Committee (reference LR20/010). Microsoft Copilot was used for language editing and improving readability only.

### 2.2. Setting

This observational study was conducted at a large publicly funded health network in Melbourne, Australia, providing acute and subacute inpatient care as well as ambulatory and community services to over 1,098,000 adults and children [29]. Occupational therapists work in a diverse range of clinical areas within the health service; more experienced staff typically work within specialty areas, whereas junior staff often work in rotational roles to gain experience in different practice areas. A typical journey for a patient with stroke would include care provided in sequence through the emergency department (usually less than 24 h); an acute inpatient ward (usually less than 1 week); a subacute inpatient ward (usually for 2–3 weeks); and finally, a community rehabilitation program (CRP), where less intensive therapy is provided at a day treatment center or within the home (usually for 6–8 weeks). There are many variations to this journey; for example, patients with mild stroke may be discharged from the acute setting, with or without a referral to CRP, or those with very severe stroke may be discharged to a residential care facility without receiving rehabilitation.

### 2.3. Participants

All occupational therapists employed at the health network who were working in stroke care were invited to participate in the survey. This included occupational therapists permanently assigned to these areas, as well as junior staff who were temporarily assigned to stroke services as part of a rotation program at the time of data collection.

Histories of all patients admitted to CRP at the health service during a 12‐month period, who had a diagnosis of stroke and were seen by an occupational therapist at any time during their acute or rehabilitation inpatient admission or while receiving follow up services in the community from the health service after discharge, were included in the file audit. CRP admissions were used as the point of recruitment as most patients with functional impairments who return to the community receive treatment in this setting by an occupational therapist. This provided a more targeted approach to reach patients who would have been more likely to benefit from somatosensory assessment and treatment while avoiding those who had a rapid and possibly more complete recovery (e.g. without the identified need for ongoing rehabilitation) or those with severe impairments discharged to residential care facilities. The file audit captured documentation across the entire duration of care for the patient with the health service spanning acute, subacute, and community episodes of care.

### 2.4. Procedure

#### 2.4.1. Part A: Survey

Occupational therapists were invited to participate in an online survey about their use of somatosensory assessments and treatments, as well as their perceived knowledge, skills, attitudes, and opportunities in this field of practice. Introductory information about the study was provided, and potential participants were prompted to sign an electronic consent form before being directed to the online survey. Study data were collected and managed using REDCap (research electronic data capture) tools [30, 31]. The questionnaire typically took about 20 min to complete.

The survey questions were based on a questionnaire developed by Cahill and colleagues [32], which included a combination of quantitative multiple choice and qualitative short answer questions. The survey explored occupational therapists′ practices relating to somatosensory assessment and treatment following stroke. Therapists were asked to indicate how often they assess and/or treat sensory impairment, and to select the assessment tools and intervention techniques they use from a list of predefined options. Respondents were also asked about factors influencing practice, including perceived barriers to providing evidence‐based care and views regarding current practice. In addition, participants rated their perceived levels of skill, skill confidence, satisfaction, and importance of sensory assessment and treatment using a scale from 0 to 100. See Table 1 for further details. We invite readers to contact authors A.J. and L.M.C. for further information or access to the survey.

**Table 1:** Summary of survey questions.

Survey domain	Content explored	Response format
Clinician characteristics	Professional background, experience, current position, and training	Multiple choice and short answer
Assessment practices	Frequency, purpose, and methods of somatosensory assessment; use of standardized and non‐standardized tools	Multiple choice, checklists, and Likert scales
Treatment practices	Frequency and type of somatosensory interventions and rehabilitation approaches used	Multiple choice, checklists, and Likert scales
Clinician perceptions	Perceived skill, skill confidence, satisfaction, importance of sensory rehabilitation, and views on current practice	Rating scales and free‐text responses
Evidence‐based practice	Sources informing practice, barriers to implementation, and workplace supports/challenges	Multiple choice and free‐text responses

#### 2.4.2. Part B: File Audit

A report of all patients referred to CRP with a diagnosis of stroke during a 12‐month study period ending in November 2020 was obtained from routinely collected hospital records. These records were then screened and accepted for inclusion if an occupational therapist had been involved in the patient′s care at any time during the patient′s stroke journey through the health service.

Evidence of somatosensory assessment and intervention was recorded if identified in any episode of care using the file audit tool (Table 2). Extracted data included demographic information, neurological diagnosis and status, physical function and rehabilitation treatments related to upper limb impairment during the patient journey, and evidence of somatosensory assessment or treatment. The auditor, an occupational therapist with expertise in stroke rehabilitation, searched for key words related to somatosensory assessment and intervention (Table 2). The inclusion of any of these words was accepted as an indication that the patient had received somatosensory assessment or treatment. Audit data were not directly and individually linked to the survey data but given the timing and setting of both sources of data, it would be expected that most of the occupational therapists treating patients in the audit sample would have also been survey respondents. See Table 2 for medical file audit variables.

**Table 2:** Medical file audit tool variables.

	Audit criteria	Response options (if applicable)
Demographics	Age	Continuous
	Length of stay (days)	Admission and discharge dates
	Gender	Female; intersex; male; not defined; other
Stroke history	Previous stroke?	Yes/no
Clinical presentation	Upper limb deficit documented?	Yes/no
Sensory assessment	Sensory assessment documented?^a^	Yes/no
	Sensory impairment evident following assessment?	Yes/no
	Standardized assessment used	Yes/no; assessment recorded
	Type of sensory assessment	Standardized or nonstandardized sensory assessment
Sensory intervention	Sensory intervention documented?^b^	Yes/no
	Type of sensory intervention	sensory discrimination/retraining, sensory stimulation/exposure, mirror therapy, desensitisation, compensatory approaches, education, proprioceptive interventions, other.

*Note:* For some variables, response options are presented in summarized form because the full set included numerous standardized assessment tools and intervention types.

^a^For this question, key terms to be observed include sensory, somatosensory, sensation, tactile, touch, temperature, proprioception, kinaesthesia, vibration, pressure, sharp/dull, two‐point discrimination, stereognosis, sensory discrimination, protective sensation, numbness, light touch, and deep pressure.

^b^For this question, key terms to be observed include sensory assessment, sensory testing, sensation, light touch, pin prick, monofilament, proprioception, dermatomes, sensory exam, sensory re‐education, sensory retraining, desensitization, sensory stimulation, tactile stimulation, graded sensory input, mirror therapy, proprioceptive training, sensory integration, sensory modulation, compensatory sensory strategies, textile exposure, texture discrimination, tactile object recognition, temperature, visual compensation, weight bearing, and desensitization.

### 2.5. Data Analysis

The data from the survey and the medical file audit were analyzed using descriptive statistics generated in Microsoft Excel [33]. These included frequencies, distributions, medians, and interquartile ranges. Where free text responses were provided, responses were coded using a content analysis approach as described by Vaismoradi et al. [34]. Responses were reviewed multiple times to become familiar with the data. Similar ideas and responses were grouped together into categories representing common themes by the first author. The number of responses within each category was then tallied to identify commonly reported concepts. Findings from the survey and file audit components of the study were compared in the discussion section.

## 3. Results

### 3.1. Participant Characteristics

#### 3.1.1. Survey Participants

A total of 15 occupational therapists, all female, responded to the survey out of a potential *n* = 22 therapists, giving a response rate of 68%. These therapists had varied roles from entry‐level therapists to senior therapists, with experience ranging from ≤ 5 years (47%), 6–10 years (20%), and greater than 10 years (33%). Of these, 12 had ≤ 5‐year experience working with patients with stroke (80%). Four respondents (27%) had undertaken additional training in somatosensory training (e.g., by attending a workshop) since graduating as an occupational therapist (Table 3).

**Table 3:** Survey respondents′ demographic information.

Demographic information: Survey respondents (*n* = 15)	Number (percentage^a^)
Degree level	
Bachelor	9 (60)
Postgraduate qualifications	6 (40)
Level of seniority	
Grade 1 (junior)	4 (27)
Grade 2 (intermediate)	7 (47)
Grade 3 (senior)	4 (27)
Years of experience working as an occupational therapist	
≤ 5	7 (47)
6–10	3 (20)
> 10	5 (33)
Years of experience working with stroke clientele	
≤ 5	12 (80)
6–10	1 (7)
> 10	2 (13)
Hours worked in typical work week	
Part time	5 (33)
Full time	10 (67)
Previously attended somatosensory training	
Yes	4 (27)
No	11 (73)

^a^Percentages rounded to nearest whole number.

#### 3.1.2. Characteristics of Patients Included in the File Audit

The patient audit sample (*n* = 50) included 29 males (58%) and 21 females (42%) with an average age of 67.9 (SD 14.7) years who were seen by an occupational therapist. Seven (14%) had experienced a previous stroke, and 26 (52%) had a documented impairment of the upper limb. The average length of stay in hospital inpatient facilities (acute and rehabilitation combined) was 18.94 (SD 24.90) days.

### 3.2. Occupational Therapists′ Use of Sensory Assessment

#### 3.2.1. Survey Results

All but one respondent (93%) felt that it was their role to assess sensation in patients with stroke. No participants responded that they never assess patients for sensory loss after stroke, but about half (*n* = 8, 53%) reported completing these assessments less than once per month. Standardized assessments were rarely used; 14 of the 15 respondents (93%) selected “none” when asked to select from a list of the most common standardized assessment tools used by Australian occupational therapists. All therapists reported that they assessed for light touch, and most reported that they assessed pressure (80%), proprioception (80%), and temperature discrimination (73%). Only a few therapists (20%) described themselves as being aware of the latest evidence in sensory assessment.

#### 3.2.2. File Audit Results

Of the 26 patients who had a documented impairment of the upper limb, 14 (54%) had a reported assessment of sensation conducted by an occupational therapist (Table 4). For those patients for whom sensation had been assessed, the audit confirmed reports by the occupational therapists that the most prevalent types of assessment were light touch (assessed in 13 patients, 93%), proprioception (assessed in 5 patients, 36%), and temperature discrimination (assessed in 4 patients, 29%). Deep pressure and kinaesthesia were assessed in one client each. The file audit provided no evidence of use of point localisation tests, texture discrimination, tactile object recognition, or pain assessments, despite therapists in the survey reporting use of these assessment techniques.

**Table 4:** Medical file audit results.

Medical file audit results	*n* (%)
Number of patients seen by an occupational therapist (*n*)	50
Demographics of those seen by OT	
Age (mean years, SD)	67.90 (14.74)
Length of stay in hospital (mean days, SD)	18.94 (24.90)
Gender (*n*, %)	
Male	29 (58)
Female	21 (42)
Other	0 (0)
Previous stroke (*n*, %)	
Yes	7 (14)
No	43 (86)
Upper limb deficit documented	
Yes	26 (52)
No	24 (48)
Sensory assessment documented	
Yes	14 (28)
No	36 (72)
Sensory impairment evident following an assessment	
Yes	12 (24)
No	38 (76)
Standardized assessment used^a^	
None	13 (93)
Monofilaments	1 (7)
Types of sensory assessments conducted	
Light touch	13 (26)
Temperature discrimination	4 (8)
Proprioception	5 (10)
Deep pressure	1 (2)
Kinesthesia	1 (2)
Sensory intervention documented	
Yes	7 (14)
No	43 (86)
Type of sensory intervention conducted	
Textile exposure	2 (4)
Texture discrimination	3 (6)
Mirror therapy	3 (6)
Tactile object recognition	2 (4)
Temperature discrimination	1 (2)
Education	1 (2)
Visual compensation	1 (2)
Deep pressure massage	1 (2)
Weight bearing	1 (2)
Desensitisation	1 (2)

^a^Percentage of the 14 patients with a documented sensory assessment.

### 3.3. Occupational Therapists′ Use of Sensory Treatment

#### 3.3.1. Survey Results

Most of the therapists (*n* = 14, 93%) felt that it was their role to treat sensory impairments in patients with stroke. However, for most respondents, sensory treatment was not frequently used, with 73% of respondents reporting using it once per month or less. When provided with a list of sensory treatment strategies and asked to select those that they did use in practice, all respondents reported using compensatory strategies (such as training patients to rely on vision instead of sensation), whereas some also reported using other treatment techniques, including repeated exposure to sensory stimuli (*n* = 12, 80%); matching or identifying textures, shapes, or objects (*n* = 7, 47%); cutaneous electrical stimulation (*n* = 5, 33%); and mirror therapy (*n* = 4, 27%).

#### 3.3.2. File Audit Results

Of the 12 patients reported to have sensory loss, treatment for sensory impairment from the occupational therapist was documented in seven cases. Components of treatment documented in the medical histories included texture discrimination (*n* = 3), mirror therapy (*n* = 3), textile exposure (*n* = 2), and tactile object recognition training (*n* = 2).

### 3.4. Factors Impacting the Provision of Evidence‐Based Care

#### 3.4.1. Capability

**Figure 1:**
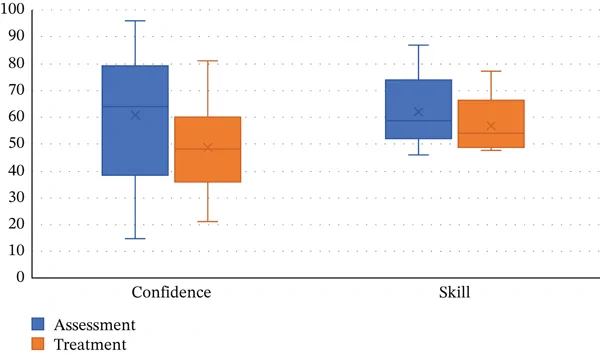
Capability: distribution, median, and interquartile ranges of self‐rated levels of skill and skill confidence in somatosensory assessment and treatment.

Therapists rated their perceived level of skill (59/100, IQR 21) and skill confidence (64/100, IQR 32) on a scale of 0–100 in sensory assessment. Their perceived level of skill in somatosensory treatment was 54/100 (IQR 19), and skill confidence was 48/100 (IQR 22) (Figure 1). Most respondents reported being unsure of best practice in this clinical area (*n* = 12, 80%) and some felt that the available research was not providing therapists with clear implications for practice (*n* = 5, 33%).

#### 3.4.2. Opportunity

Occupational therapists reported a range of opportunity barriers to sensory assessment and treatment in clinical practice. A lack of time (*n* = 12, 80%) was the most reported barrier. Small numbers of participants (ranging from 1 to 3 respondents) reported experiencing a range of other suggested barriers, such as access to research literature, limited resources to support change, or training barriers.

#### 3.4.3. Motivation

**Figure 2:**
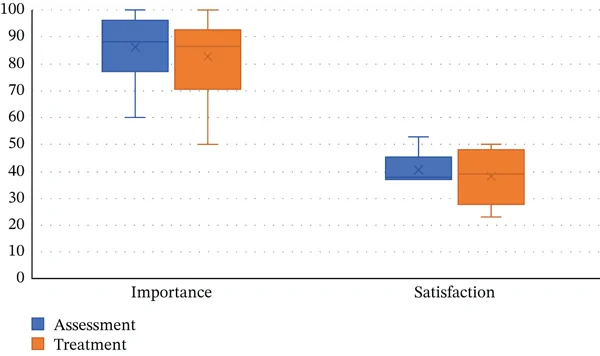
Motivation: distribution, median, and interquartile ranges of self‐rated levels of importance and satisfaction with somatosensory assessment and treatment.

When asked to rate the importance of the assessment of sensation on a scale of 0–100, the median rating of 88 (IQR 17) suggests that occupational therapists perceive this to be highly important. Respondents had similar attitudes to treatment of sensation (mean rating 86.5/100, IQR 16.25). However, occupational therapists had mid‐range overall satisfaction with their current practice both in relation to the assessment (median 43/100, IQR 20) and treatment (median 41/100, IQR 15.5) of sensation (Figure 2).

#### 3.4.4. Behavior Change

When asked whether they would like to make changes to their current practice, most respondents (*n* = 11, 73%) expressed a desire to change practice in somatosensory assessment, and all but one respondent (*n* = 14, 93%) expressed a desire to change their current practice in somatosensory treatment. However, the use of research evidence was reported by some to not be a high priority within the workplace (*n* = 5, 33%).

## 4. Discussion

Findings from this research provide evidence from two sources (survey and file audit) that occupational therapists working in a variety of clinical settings within a large metropolitan health service in Australia infrequently assess and treat somatosensory impairments in stroke survivors and use a relatively narrow range of non‐standardized assessment and treatment methods. Despite occupational therapists in this study seeing sensory assessment and treatment as important and part of their clinical role, they rated their skill and skill confidence as low. Nearly half (42%) of people who were identified as having sensory loss missed out on receiving sensory treatment.

Evidence‐practice gaps in somatosensory treatment are not new [24]. Previous studies have found significant gaps between evidence and practice, with occupational therapists tending to favor compensatory strategies over evidence‐based treatment techniques or failing to provide treatment for sensory impairments [24, 35]. In our sample, almost half of patients with sensory loss did not receive somatosensory treatment based on audited case notes, despite evidence‐based somatosensory interventions being available [16, 36]. Despite advancements in the literature supporting somatosensory assessment and intervention [17, 37], it appears that the generation of this evidence remains insufficient to change routine clinical practice, at least in the health service investigated. The persistence of this evidence‐practice gap highlights the complexity of implementation in rehabilitation settings where care delivery is influenced by competing priorities, including service constraints and opportunities for therapists to improve their knowledge and skills [38]. Understanding these factors is needed to design implementation strategies that can support evidence‐based care delivery.

Occupational therapists expressed low rates of satisfaction and skill in this area of practice, suggesting a gap in capability consistent with previous findings [24]. Most participants in the current study agreed that their current practice in both sensory assessment and treatment for stroke survivors needs to be improved, suggesting that strategies that address capability may enhance the uptake of evidence‐based practice in this field. Capability strategies could include targeted professional education, coupled with local strategies to support clinicians to build skill confidence and integrate new learning into practice, including audit and feedback, peer mentoring, and leadership support [39].

Although sensory assessment and treatment were identified as being important, occupational therapists may have limited opportunity to use these skills on a regular basis with roughly half of clinicians reporting to assess sensation less than once per month. However, evidence indicates that somatosensory impairment is present in about 50% of stroke survivors and has a negative impact on motor function and participation in daily activities [6, 7]. We do not know definitively what the cause of the low rates of assessment in this study are due to. What we do know is that this issue is twofold: clinician level and client level. In stroke rehabilitation, regular use of assessment and interventions is correlated with increased clinician confidence and capability [39, 40]. With the current assessment frequency, there is both a gap in building clinician confidence and the provision of evidence‐based care. Without regular use of these assessments, clients affected by stroke with somatosensory impairment will continue to miss out on evidence‐based care.

Participants reported no use of standardized assessments. In contrast, assessments that could be completed without specialized equipment, such as light touch and proprioception testing, were used regularly. This observation might reflect the barriers identified by this study including limited time, access to research, insufficient resources to support change, and barriers to training. Assessments that require specialized equipment, such as temperature assessment or adherence to an assessment administration protocol, may be more difficult to integrate into routine practice. These findings are consistent with previous research that highlights time, resources, and training demands as barriers to implementing evidence‐based practice [24, 41]. A notable exception was object recognition retraining. Despite no reports of assessing object recognition, this intervention was used frequently. One possible explanation is that the required equipment had been available within the department for a long time and clinicians were already confident and familiar with its use, reducing barriers to incorporation into routine practice [40]. These findings suggest that assessment and interventions that are supported by protocols for delivery or require the use of specialized equipment need to be embedded into routine care processes. Having easy access to equipment and clinical guidance, such as prompt sheets for delivery, may reduce barriers to the implementation of evidence‐based practice, given the multiple competing role demands within inpatient rehabilitation settings for occupational therapists [42].

The proportion of patients documented as having somatosensory impairment in this study was relatively low (46% of stroke survivors with upper limb impairment). Given that previous studies suggest between 50% and 80% of people affected by stroke experience somatosensory impairment [43], this suggests this finding may reflect under‐recognition or under‐documentation of somatosensory impairment, rather than a truly lower prevalence. To explore these issues, the study included all patients seen by an occupational therapist with a diagnosis of stroke rather than restricting inclusion to only participants with identified upper limb or somatosensory impairment. Restricting the sample to patients with identified upper limb or somatosensory impairment assumes that impairments were consistently identified and documented, limiting the ability to identify potential gaps in assessment practices. A study limited to people with identified somatosensory loss could provide more detailed observations of treatment approaches; however, the broader inclusion criteria used in this study allowed observation of real‐world clinical behaviors.

The third domain that could explain evidence‐practice gaps in this field is the motivation of occupational therapists. Within the COM‐B model, motivation is comprised of automatic motivation (including impulses and inhibitions) and reflective motivation (processes involved in making plans) [27, 44]. Findings relating to motivation were mixed. Most rated this clinical area as important and expressed a desire to change their practice suggesting relatively high automatic motivation. However, approximately one third of survey respondents reported that the use of research evidence was not a high priority. This finding is consistent with a previous focus group study conducted at this same health service, which found that evidence‐based practice was sometimes perceived as secondary to other clinical demands by allied health therapists or managers [41]. Although motivational barriers were evident for some participants, barriers related to opportunity, particularly access to resources and research literature, were reported more consistently. In this context, organizational strategies that improve access to evidence and support engagement with research may be more effective in translating motivation into practice change.

Medical record data audited in this study largely supported self‐reported survey data, but there were two main areas of divergence. Firstly, audit of patient files indicated that occupational therapists overestimated the frequency with which they used sensory assessment and treatment during their practice, as reported in their survey responses. Secondly, occupational therapists reported assessing a greater number of sensory domains in the survey than what was reflected in documentation in medical records.

There are several possible explanations for these discrepancies. One possibility is that occupational therapists may not be documenting all areas of sensory assessment and treatment in the medical file. This is supported by the relatively low number of patients with upper limb deficits who were documented as having a somatosensory deficit despite an estimated prevalence of sensory changes among stroke survivors of 50% [43]. In addition, therapists might recall having done the sensory assessment or treatment but have some recall bias about how frequently this took place. Furthermore, there could be an element of social desirability bias where survey respondents were trying to give the “right” answer rather than reporting what they actually do [45]. This divergence highlights the limitations of self‐report data alone for understanding alignment between evidence and practice.

These findings suggest that people affected by stroke are missing opportunities to receive evidence‐based rehabilitation for this reasonably common poststroke impairment, with implementation strategies needed to target clinician capability and confidence. A change in clinical practice can only occur when factors that are acting as barriers to change have been acknowledged and addressed [27]. Automatic motivation appears to be present for the therapists in this study and could be harnessed to build a case for change. The findings of the current study support the use of strategies aiming to increase knowledge, skill, and confidence as a foundational first step to improve the use of evidence‐based care in somatosensory assessment and treatment, addressing the capability aspect of the COM‐B model [27].

However, previous research has shown that knowledge change alone is often insufficient to bring about a change in practice [22, 46]. To be more comprehensive in supporting a change in behavior, capability interventions may need to be supplemented with implementation strategies focused on creating opportunity for occupational therapists to implement evidence into practice. Further research is needed to identify and test innovative solutions that support the integration of somatosensory assessment and intervention into routine clinical practice. This includes creating practical and realistic strategies to overcome challenges related to capability, opportunity, and motivation within complex and busy clinical settings.

Strengths of this study include the use of two data sources to triangulate clinician‐reported findings with documented therapy provided. This study also provides a template for investigating baseline practice as part of a site‐specific implementation study, with the example being a large health network with acute inpatient rehabilitation and multiple community services. One advantage of using the previously published survey is that it permits benchmarking of results in future [34], whereas review of patient files ensures that the baseline data sit within site‐specific organizational practice and expectations. Moreover, this site‐specific approach supports tailored implementation, as recommended in known behavior change frameworks [26, 27]. The use of an implementation framework, the COM‐B is a strength of the study, providing a useful framework for identifying broad influences on implementation within the domains of capability, opportunity, and motivation [27].

Limitations to this study include the observational study design, a relatively small sample size, a lack of data around the participants′ role in acute rehabilitation or community services, and the retrospective nature of the audit and survey questions, which can all lead to potential bias in the findings. Additionally, medical file audit data describes “work documented” and is no guarantee of “work done” but still offers an alternative source of data that is valuable as a supplement to self‐reported data provided by clinicians. Inclusion of both a clinician questionnaire and a medical file audit increases the credibility of the findings. Of all the patients included in the file audit, only a small group of patients had documented somatosensory impairment, which limits the strength of the conclusions regarding somatosensory treatments. However, this also highlights an important finding of the study that assessment of somatosensory function appeared to be completed inconsistently. This study took place at one health service, where the findings are influenced by contextual factors specific to the service. Given the myriad of literature around the impact of context on clinical practice, these findings should be interpreted with caution when generalizing to other service contexts [47, 48]. However, these contextual variations may provide opportunities for health services to learn from one another, and findings could be useful as a foundation for other contexts.

## 5. Conclusions

Findings from this study highlight that a practice gap continues to exist in the use of evidence‐based somatosensory assessment and treatment by occupational therapists. This study provides a unique perspective by triangulating clinician perceptions (survey) with clinical file audit data. Most occupational therapists recognized the importance of sensory assessment and therapy; however, they reported barriers in both capability (lack of skills) and opportunity (a lack of time and limited opportunity to practice their skills) impacting evidence‐based practice in this clinical area. Encouragingly, occupational therapists expressed motivation to change their current practice and improve their skills in both somatosensory assessment and treatment, suggesting that motivation is unlikely to be the primary barrier to change.

These findings have implications for education, clinical practice, behavior change, and future research. Efforts to improve alignment between evidence and practice in stroke rehabilitation should focus on developing capability and confidence, as well as tailored strategies to facilitate opportunity, such as protected learning time, access to resources, opportunities for practice, and feedback. Future research could evaluate whether these tailored implementation strategies in conjunction with educational endeavors improve capability, confidence, and evidence‐based care delivery.

## Author Contributions

All listed authors provided substantial contributions to the conception and design of the work and the acquisition, analysis, and interpretation of data; they drafted or revised the work critically for important intellectual content.

## Funding

This work was supported by the National Health and Medical Research Council, 10.13039/501100000925, GNT1134495 and GNT2004443. Open access publishing facilitated by Monash University, as part of the Wiley ‐ Monash University agreement via the Council of Australasian University Librarians.

## Disclosure

All authors meet the ICMJE criteria, provided final approval of the version to be published, and were in agreement to be accountable for all aspects of the work in ensuring that questions related to the accuracy or integrity of any part of the work are appropriately investigated and resolved.

## Conflicts of Interest

The authors declare no conflicts of interest.

## Data Availability

The data that support the findings of this study are available from the corresponding author upon reasonable request.
